# Postadmixture Selection on Chileans Targets Haplotype Involved in Pigmentation, Thermogenesis and Immune Defense against Pathogens

**DOI:** 10.1093/gbe/evaa136

**Published:** 2020-07-02

**Authors:** Lucas Vicuña, Olga Klimenkova, Tomás Norambuena, Felipe I Martinez, Mario I Fernandez, Vladimir Shchur, Susana Eyheramendy

**Affiliations:** e1 Faculty of Engineering and Sciences, Universidad Adolfo Ibañez, Peñalolén, Santiago, Chile; e2 National Research University Higher School of Economics, Russian Federation, Moscow, Russia; e3 Center for Intercultural and Indigenous Research, School of Anthropology, Faculty of Social Sciences, Pontificia Universidad Católica de Chile, Santiago, Chile; e4 Department of Urology, Clínica Alemana, Santiago, Chile; e5 Center for Genetics and Genomics, Faculty of Medicine, Clínica Alemana, Universidad del Desarrollo, Santiago, Chile; e6 Instituto Milenio de Investigación sobre los Fundamentos de los Datos (IMFD)

**Keywords:** adaptation, genetic ancestry, admixture, gene flow, genetic drift

## Abstract

Detection of positive selection signatures in populations around the world is helping to uncover recent human evolutionary history as well as the genetic basis of diseases. Most human evolutionary genomic studies have been performed in European, African, and Asian populations. However, populations with Native American ancestry have been largely underrepresented. Here, we used a genome-wide local ancestry enrichment approach complemented with neutral simulations to identify postadmixture adaptations underwent by admixed Chileans through gene flow from Europeans into local Native Americans. The top significant hits (*P *=* *2.4×10^−7^) are variants in a region on chromosome 12 comprising multiple regulatory elements. This region includes *rs12821256*, which regulates the expression of *KITLG*, a well-known gene involved in lighter hair and skin pigmentation in Europeans as well as in thermogenesis. Another variant from that region is associated with the long noncoding RNA *RP11-13A1.1*, which has been specifically involved in the innate immune response against infectious pathogens. Our results suggest that these genes were relevant for adaptation in Chileans following the Columbian exchange.

## Introduction

Among other evolutionary forces such as positive selection and drift, genetic admixture has been shown to play an important role in shaping human diversity (Nielsen et al. 2017). The majority of human groups studied to date experienced ancient (<7,000 years) to recent (<500 years) admixture, whereas a limited number of populations have remained in isolation for longer periods (Pickrell and Reich 2014). Selective pressures exerted long enough over populations as well as genetic drift generate regional differences in allele frequencies (Coop et al. 2010), whereas admixture tends to neutralize these differences through gene flow (Pickrell and Reich 2014). However, standing genetic variation inherited by the admixed individuals can sweep into high frequency over generations if this variation aids them to adapt to endemic or foreign selective pressures. This *post-admixture* selection (hereafter referred to as “PAS”) is similar to adaptive introgression at the intraspecies level (Bhatia et al. 2014; Jeong et al. 2014).

Some studies have shown that PAS underlies adaptation in diverse human populations. For instance, modern high-altitude Tibetans, who resulted from the admixture of populations ancestral to the high-altitude Sherpa and low-altitude Han Chinese, underwent PAS in the *EGLN1* and *EPAS1* genes. These genes are important components of the hypoxia-inducible pathway, which is involved in changes in O_2_ supply and is thus relevant for breathing at high altitudes (Jeong et al. 2014). In addition, the African-specific *Duffy-null* blood group—which confers resistance against *Plasmodium vivax* infection—underwent PAS in the Makranis of Pakistan after it was introduced by sub-Saharan African slaves into Pakistan (Laso-Jadart et al. 2017). Among admixed Mexicans, PAS acted on the major histocompatibility complex region, as revealed by significant departures of African ancestry in that genomic region (Guan 2014). Similarly, a study used microsatellite data from a set of pulled Latin American populations with Native American, European, and African ancestry to detect PAS. The top signals were located in gene regions with important roles in immune defense, including the HLA region (excess of African ancestry) and the immunoglobulin heavy chain gene complex region (excess of European ancestry). This suggests that the corresponding genes were relevant for adaptation to infectious diseases brought by nonnative immigrants (Deng et al. 2016). PAS acting on Native American genetic variation in lipid-related genes was associated with a higher incidence of obesity and dyslipidemias in admixed Mexicans (Ko et al. 2014). Similarly, a study on admixed Brazilians found PAS acting on a Native American haplotype encompassing the *PPP1R3B* gene, which is involved in glycogene synthesis. Such variants in Mexicans and Brazileans may have been advantageous due to increased fat and glucose storage under a restrictive diet environment (Secolin et al. 2019; Ko et al. 2014). Further, a recent study found that PAS acting on Native American genetic variation may have aided a population with Native American and European ancestry from the Atacama Desert of Northern Chile to adapt to the pathophysiological effects of chronic exposure to high arsenic levels in water (Vicuna et al. 2019).

The Chilean population is a suitable model for analyzing how PAS has acted through gene flow from Europeans into Native Americans. Chileans have similar proportions of European and Native American ancestry (0.52 and 0.45, respectively) and a small African component (0.03) (Eyheramendy et al. 2015). These proportions resulted from an admixture process that began in the 16^th^ century with European settlers (mostly Spaniards) who admixed with Native Americans from the lowlands of Central-Southern Chile (the Mapuche) and the Andes highlands of Northern Chile (the Aymara). The admixture process continued with several minor migratory events at later times (mainly from Europe), including a small African component brought in during the 17^th^ century (Eyheramendy et al. 2015).

The aim of this study was to evaluate whether Europeans provided adaptive genetic variation to admixed Chileans by acting through PAS following the Columbian exchange. We analyzed this selective force in a population from the Atacama Desert of Northern Chile (Vicuna et al. 2019). We identified significant PAS signatures at a haplotype rich in regulatory elements. This haplotype included *rs12821256*, an enhancer variant that regulates the expression on *KITLG*. Although *rs12821256* is a causative variant of lighter hair color in northern Europeans (Guenther et al. 2014), *KITLG* regulates the amount of melanin pigments in hair follicles as well as in the skin and is also involved in thermogenesis (Yang et al. 2018). *KITLG* variants, including *rs12821256*, have undergone strong positive selection in Europeans (Grossman et al. 2010; Yang et al. 2018). The selected haplotype also includes a variant associated with the long noncoding RNA (lncRNA) *RP11-13A1.1*, which has been specifically implicated in the immune response against fungal infections (Riege et al. 2017). Our results suggest that these regulatory elements were relevant for adaptation among admixed Chileans following the Columbian Exchange.

## Materials and Methods

### Genomic Data

We used whole-genomes from 11 Huilliche/Pehuenche individuals from Vidal et al. (2019), which belong to the macroethnic Mapuche population. Whole-genome data were taken from 108 Yoruba in Ibadan, Nigeria (YRI); 107 Iberian Population in Spain (IBS); 107 Toscani in Italy (TSI); 99 Utah Residents with Northern and Western European Ancestry (CEU); 91 British in England and Scotland (GBR); and 99 Finnish in Finland (FIN) individuals from the 1000 G Project (1000 Genomes Project Consortium et al. 2015). We used SNP array data of 25 Aymara, 24 Quechua, 25 Maya, and 14 Nahua Native Americans from Bigham et al. (2010). We used an SNP array data set from a previous study (Vicuna et al. 2019) consisting of 185 admixed subjects (121 males and 64 females) with mostly European and Native American ancestry from the Atacama Region of Northern Chile. We performed the same quality controls as in Vicuna et al. (2019). Briefly, using Plink 1.9 (Purcell et al. 2007), we excluded genetic variants with minor allele frequency <0.01, SNP calling rate <90%, individual calling rate <90%, and Hardy–Weinberg Equilibrium *P* value <0.00001. We obtained a final set of 895 individuals (including the 185 Chileans) and 772,277 SNPs.

### Local Ancestry Inference

We estimated local ancestry in Chileans at these 772,277 SNPs using LAMP-LD 1.1. We used reference panels for Native American (*n* = 88), European (*n* = 911), and African (*n* = 229) populations, as described in Vicuna et al. (2019). LAMP-LD uses a Hidden Markov Model (HMM) that integrates out all possible phase switches in the admixed genotypes when estimating the total genotype probability. To speed up computation, only phase switches that change ancestry (i.e., breakpoints) are constrained to occur at boundaries of windows of L SNPs; these breakpoints are subsequently further resolved in a final step using a simplified HMM that allows a single ancestry switch for local region around that breakpoint (Baran et al. 2012). We used windows of size *L* = 10 and an HMM with number of hidden states *S* = 15. We used phased samples from the parental populations and unphased data from the Chilean population. Phasing was performed using Beagle (Browning and Browning 2007). As readout, LAMP-LD produces individual chromosome files where each SNP allele is coded as 0, 1, or 2, depending on whether the haplotype block harboring the SNP has a higher probability of being of European, Native American, or African ancestry, respectively. After filtering only autosomal SNPs with known rs IDs, we obtained a final data set of 633,744 SNPs.

### Global Ancestry Inference

We selected autosomal SNPs that intersect between all the population data sets and then merged the 12 populations using Plink. We further excluded SNPs with SNP calling rate <95% to the merged file, obtaining a final set of 420,688 SNPs and 895 individuals. We used the *Ohana* software suite (Cheng et al. 2017) to infer the individual ancestry components of our Chilean population. For this, the clean data set was converted to .dgm format with *Ohana* using convert ped2dgm. Then, the converted data set was down-sampled to 5% of the number of SNPs (sampled randomly) using the sample-sites.py script provided in the *Ohana* GitHub repository https://github.com/jade-cheng/ohana (Cheng et al. 2017). In this way, we obtained a high-quality subset of ∼100,000 unlinked markers to use for the structure analysis, as recommended in that repository. Then, qpas -e 0.0001 -k i -qo was used to create Q matrices with the likelihood of the ancestral component proportions, where i represents the number of ancestry components and ranges from 3 to 10. Admixture plots were plotted using pong (Behr et al. 2016). ADMIXTURE was used to compute the cross-validation error among different Ks (Alexander et al. 2009). For each value of *K*, ten replicates were performed, and the *K* with the lowest mean cross-validation error was assumed to have the best predictive accuracy.

### Principal Component Analysis

**Fig. 1.**
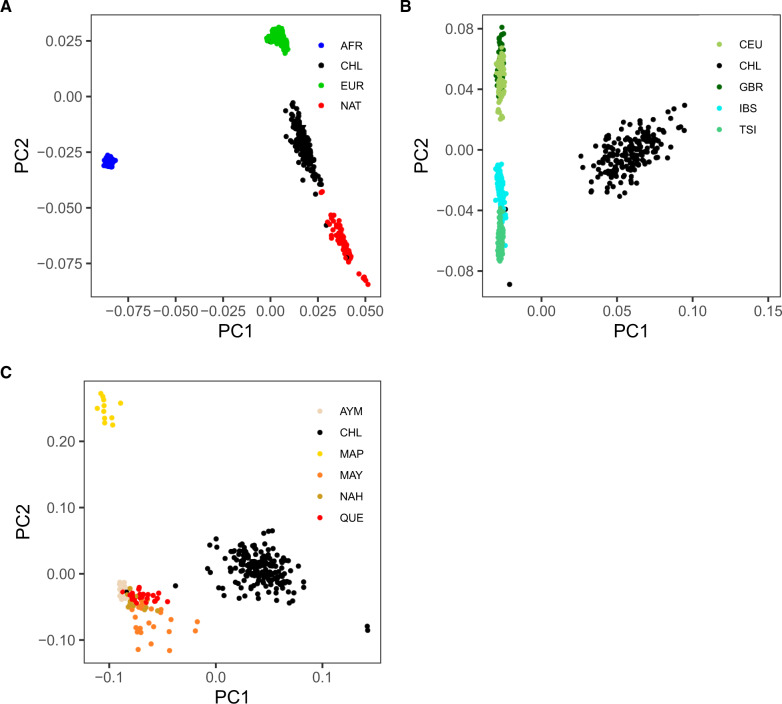
Principal component analysis. Principal component clustering of admixed Chileans together with populations from Europe, the United States, and Africa. (*A*) PC1 versus PC2 of Chilean (CHL), European (EUR), Native American (NAT), and African (AFR) populations. Populations included are: CEU, GBR, IBS, TSI [Europe]; Aymara (AYM), Mapuche (MAP), Maya (MAY), Nahua (NAH), Quecha (QUE) [the United States]; and YRI [Africa]. (*B*) PC1 versus PC2 of Chileans and CEU, GBR, IBS, and TSI. (*C*) PC1 versus PC2 of Chileans and the five Native American populations.

Principal component (PC) analysis on the clean data set of 420,688 SNPs and 796 individuals (FIN samples were excluded from the original data set) was carried out with Plink, using the following filters to obtain an LD-pruned subset of variants. We only included variants with minor allele frequency >0.01, SNP calling rate >99%, and we pruned SNPs using an independent pairwise approach with window size of 50 kb, a step size of five SNPs, and a *r*^2^ cutoff threshold of 0.15. Eigenvectors were produced with Plink. PC plots were generated with R. The PCs from figure 1*B* and *C* were obtained using the same filters, but excluding all Native American and European samples, respectively.

### Deviations from Mean Genome-Wide Local Ancestry

Following local ancestry estimations with LAMP-LD (see Local Ancestry Inference), we calculated the mean local ancestry for every SNP using an in-house R script (supplementary file 1, Supplementary Material online). We used one-tailed *t*-tests to identify SNPs with deviations in the mean European ancestry. At each SNP, we compared its European ancestry proportion with the genome-wide ancestry mean *p*_0_=0.52. We performed a statistical hypothesis test *H*_0_: *p*_1_=*p*_0_ versus *H*_1_: *p*_1_ > *p*_0_ at each SNP, where *p_i_* represents the European ancestry at the *i*th SNP. We assumed that *X*_ij_ is a random variable following a Binomial distribution with parameters *n *=* *2 and *p_i_*, where *X*_ij_ takes values in {0,1,2} representing the number of European alleles at SNP i and individual j. *p_i_* is the proportion of European ancestry at SNP i. The asymptotic results of the maximum likelihood estimator for *p_i_* were used to assume a normal distribution on this estimator and to design our hypothesis test. Variants reaching a significance threshold of *P *<* *10^−5^ were considered to be under PAS (Bhatia et al. 2014).

### Estimation of the Effective Population Size of Chileans

We estimated the effective population size (*N*_e_) of our Chilean sample as follows. The initial ancestry proportion *p*_0_ of European component changes over time due to genetic drift. At the sampling time, the European proportion *p*_1_ is a random variable with the variance approximately equal to (Tataru et al. 2017):

$$\mathrm{var}=p_{0}\left(1-p_{0}\right)\left(1\;-\;e^{-\frac{T}{2Ne}}\right).$$

The expectation of *p*_1_ equals the admixture proportion *p*_0_, and the distribution of *p*_1_ can be approximated by a Beta distribution with the mean and variance equal to these values. Next, we took into account that the European ancestry observed in our empirical data depends on the sampling. At each SNP, the observed proportion *k* of European ancestry was sampled from the binomial distribution with sample size K(in our case K = 370) and probability *p*_1_. So, the observed European ancestry is distributed as follows:

$$k\;\sim \;\int_{0}^{1}P\left(\mathrm{Binom}\left(p_{1},\;K\right)=\;k\right)d\left(p_{1}\right).$$

We adjusted *N*_e_ so that the variance of *k* matches the empirical variance of European ancestry across all the SNPs in our Chilean sample of size *N* = 370 haplotypes. For different times since admixture, we got the following values for the effective population sizes: *N*_e_ = 4,500 at *T* = 10, *N*_e_ = 6,000 at *T* = 12, and *N*_e_ = 7,000 at *T* = 15.

### Simulations of Local Ancestry

We performed forward-time local ancestry simulations using SELAM (Corbett-Detig and Jones 2016). We assumed a scenario with a single pulse of admixture of three populations *T* generations ago (setting *T* to three different values of 10, 12, and 15 generations). The admixture proportions were set to 0.521 (European), 0.442 (Native American), and 0.037 (African), as those estimated by LAMP-LD in our Chilean sample. We assumed the *N*_e_ of the admixed population to be constant. We explored three different diploid (haployd) sample sizes: *N* = 93 (186), 185 (370), and 370 (740). For the case of 185 individuals, we simulated sexual populations (64 females and 121 males), whereas for the other sample sizes, we simulated hermaphroditic populations. We simulated 189 chromosomes each of length = 20 Morgans, so that the total length of the simulated genomes is equal to the length of our data. Then we mapped the physical positions of the SNPs in our data on the simulated chromosomes, which gives the same correlation structure in the SNP ancestry as in the real data. We calculated *t*-test *P* values for the SNP with maximal European ancestry deviations for different combinations of *N*, *N*_e_, and *T* (Table 1), similarly as indicated in supplementary file 1, Supplementary Material online.

### Variant and Gene Annotations

All variant annotations used in this study corresponded to the GRCh37 (hg19) assembly. Variant annotations including the Sequence Ontology (SO) consequence type, associated alleles, Gencode biotypes, and combined annotation dependent depletion (CADD) scores were retrieved using the web tool Variant Effect Predictor (VEP) from Ensembl (McLaren et al. 2016). Downstream variants were defined as those located within 10 kb downstream of a particular gene. Expression quantitative trial loci (eQTL) associations were retrieved with the HaploReg web tool (Ward and Kellis 2012). We used the GWAS Catalog (Welter et al. 2014) to identify phenotypes that have been associated through GWAS with our selected variants and genes.

## Results

### Identification of the Main Ancestral Components of Admixed Chileans

We first estimated genetic affinities between the admixed Chilean population from the Atacama Desert and their proxy parental populations from Europe, the United States, and Africa by PCs clustering (fig. 1*A*). From Europe, we included Iberians (IBS), Italians (TSI), Utah residents with European ancestry (CEU), and British (GBR) populations (1000 Genomes Project Consortium et al. 2015). From Native America, we used genotype data from Mapuche (Vidal et al. 2019), Aymara, Quechua, Nahua, and Maya (Bigham et al. 2010). From Africa, we used the Yoruba (YRI) population (1000 Genomes Project Consortium et al. 2015) as a proxy population for the small (∼3%) African component of Chileans (Eyheramendy et al. 2015). The final merged data set consists in 796 individuals. (See Materials and Methods for the details of the populations included in the analysis.) As expected, Chileans are scattered between the European and Native American samples, with Africans farther away (fig. 1*A*). Because sample size differences may distort the PCA (McVean 2009), we analyzed an equal number of random Chilean, Native American, European (IBS), and African (YRI) individuals (*n* = 99×4 = 396; supplementary fig. 1, Supplementary Material online). We find that the PCA results obtained using unequal and equal sample sizes are consistent between each other (fig. 1*A* and supplementary fig. 1, Supplementary Material online).

To test for affinities between Chileans and Europeans, we performed a second PCA considering only Chileans and the aforementioned European populations (total sample size = 476). We find that IBS is the most closely related European populations to Chileans, as it can be seen along the PC2 axis in figure 1*B*. This is in agreement with previous studies (Eyheramendy et al. 2015).

To evaluate genetic relationships between Chileans and Native Americans, we performed a third PCA comparing Chileans with the aforementioned Native American populations (total sample size = 284). We find that the Aymara and Quechua cluster closest to Chileans, as revealed by the PC2 axis of figure 1*C*. This finding is in line with the historical geographic range of the Aymara and Quechua in The Andes of Northern Chile, Southern Perú, and Southeastern Bolivia (Browman 1984). The Mapuche are the major Native American population of Chile. Unexpectedly, we found that besides clustering farther away from Chileans than the Aymara and Quechua along the second PC, the Mapuche cluster farther than Maya and Nahua Native Mesoamerican populations as well. This may be a distortion caused by a very low genetic variation of the Mapuche sample that resulted from at least two strong population bottlenecks experienced by them in the last centuries (see Discussion).

### Global Ancestry Estimation of Admixed Chileans

**Fig. 2.**
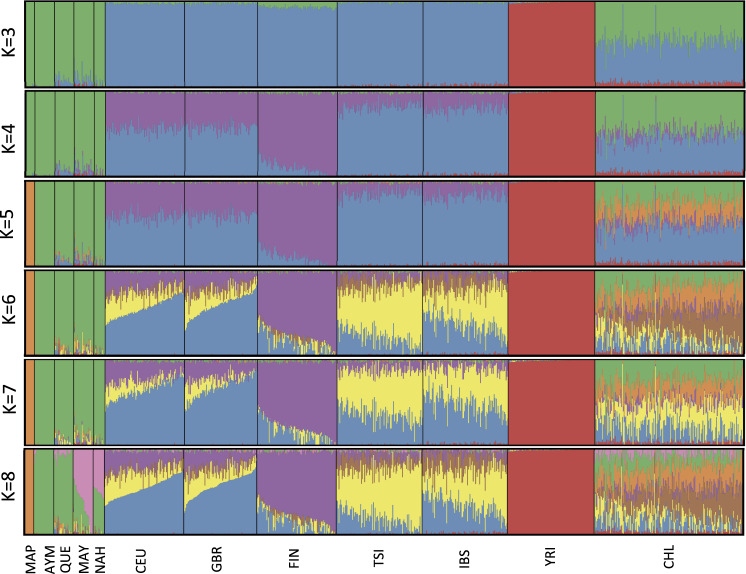
Global ancestry proportions of Chileans, European, Native American, and African populations. Cluster analysis generated by *qpas* for Chileans as well as a set of Native American, European, African, and Chilean populations. The number of displayed clusters is *K* = 3–8.

We estimated global ancestry proportions of our Chilean sample. For this, we used the program *qpas* from the *Ohana* suite software (Cheng et al. 2017), which uses an unsupervised algorithm to estimate global ancestry components based on the allele frequencies of proxy populations for individual ancestry components. Using *K* = 3, *qpas* clearly separates Chileans into European, Native American, and African ancestral components (fig. 2). *K* = 4 clusters the European populations into two main ancestral components; one more enriched in Southern European populations (IBS and TSI; fig. 2, in blue), and another more enriched in Northern European populations, particularly in FIN (fig. 2, in purple). Chileans have mostly the Southern European component, in line with our PCA results. The main Native American components of Chileans are Mapuche and Aymara, with an average Chilean having 40% Mapuche and 8% Aymara ancestry, respectively (Lorenzo Bermejo et al. 2017). To get a better estimate of these proportions in our cohort from Northern Chile, we run *qpas* using *K* = 5, which showed the best predictive accuracy across *K* = 3–12, given its lowest cross-validation error (CV error = 0.457) inferred with ADMIXTURE (Alexander et al. 2009). We could identify Aymara and Mapuche as the main Native American subancestry components of admixed Chileans from Atacama, as revealed when contrasted with the reference native Mapuche and Aymara groups (fig. 2, in orange and green, respectively). *qpas* (*K* = 5) estimates mean ancestry proportions of 20.7% Mapuche and 25.9% Aymara, 43.3% Southern European, 6.5% Northern European, and 3.5% African among Chileans. When summing up the contribution of each of these subancestry components, we obtain global European, Native American, and African proportions of 49.8%, 46.6%, and 3.5%, respectively. These estimates are slightly different to the genome-wide local ancestry means estimated by LAMP-LD (52.1%, 44.2%, and 3.7%, respectively), probably reflecting differences between the underlying algorithms. Of note, most populations included in this study have slight traces of other ancestry types. For instance, IBS and TSI have 1.5–1.7% African proportions, whereas FIN shows 6.3% of an ancestral component related with Native American populations (fig. 2). This latter observation possibly reflects ancient Siberian gene flow into the Finnish population (Lamnidis et al. 2018) and Native Americans (Nielsen et al. 2017). *K* = 6–7 identifies separate clusters within the Southern and Northern European components (fig. 2, in yellow and brown, respectively). *K* = 8 differentiates between Mesoamerican (Maya-Nahua) and Andean (Aymara-Quechua) subancestry components (fig. 2, in pink, green, and orange, respectively).

### Postadmixture Adaptation Mediated by Gene Flow in Chileans

**Fig. 3.**
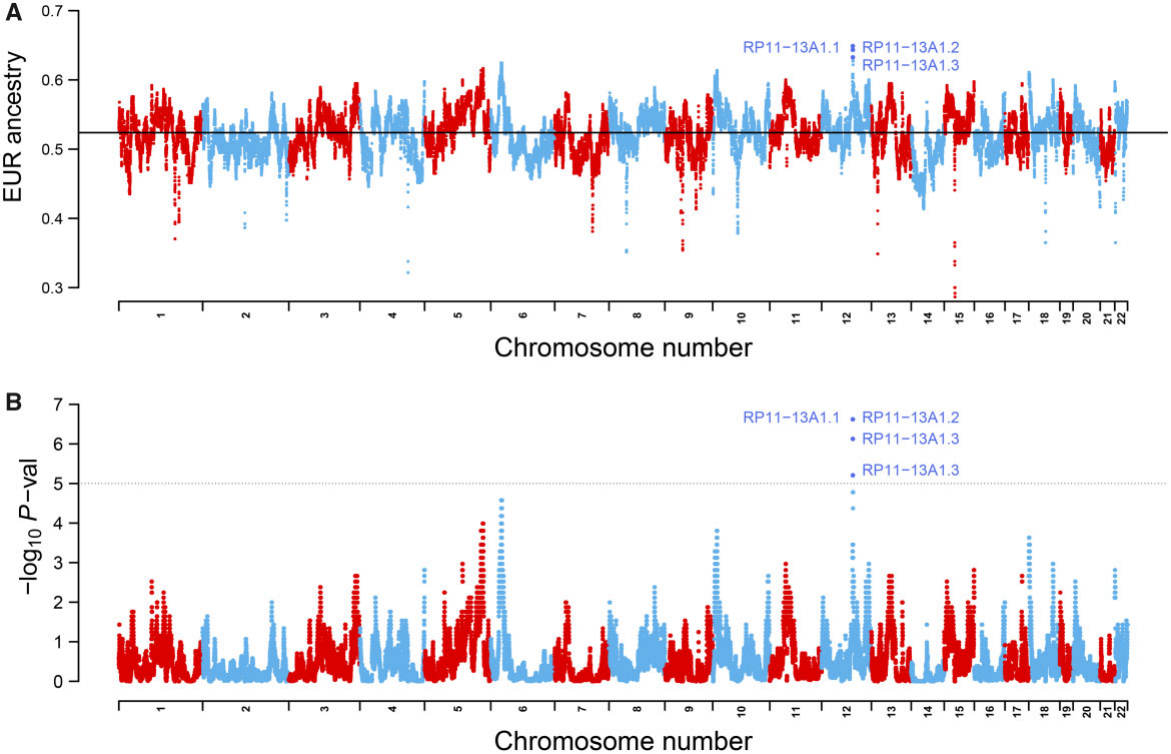
Genome-wide deviations in the local mean European ancestry among Chileans. (*A*) Deviations in the mean local European ancestry over the genome-wide mean along autosomes. The black horizontal line represents the genome-wide local European ancestry mean (0.521) (*B*). *t*-Test *P* values of the deviations expressed as −log_10_*P* value. The dotted black line represents the cutoff threshold of *P *<* *10^−5^ for SDs (Bhatia et al. 2014). Highlighted are genes associated with variants significantly enriched in European ancestry.

We hypothesized that admixed Chileans underwent PAS acting on European standing genetic variation (PAS acting on Native AMerican variation in this population was analysed in Vicuna et al. (2019)). PAS can be identified by detecting SNPs with SDs in the mean local ancestry from the genome-wide mean (Bhatia et al. 2014; Jeong et al. 2014). At each SNP, the mean European ancestry was compared with the genome-wide ancestry mean 0.52. Using *t*-tests, we evaluated the hypothesis *H*_0_: μ_EUR, i_ = 0.52 versus *H*_1_: μ_EUR, i_ > 0.52, at each variant i. We found 85 SNPs reaching the statistical significance threshold of *P *<* *10^−5^ recommended for recently admixed populations (Bhatia et al. 2014). Figure 3*A* shows the mean European ancestry at each SNP along autosomal chromosomes, highlighting selected genes. Figure 3*B* shows the corresponding *t*-test *P* values. Supplementary table 1, Supplementary Material online, contains a list of variants showing an excess of European ancestry as well as the corresponding association *P* values. The 85 SNPs map a peak of European ancestry in chromosome 12 associated with several regulatory regions, including two lncRNAs (*RP11-13A1.1* and *RP11-13A1.3*) and one processed pseudogene (*RP11-13A1.2*; supplementary table 1, Supplementary Material online).

To evaluate whether these extreme deviations in local ancestry are due to genetic drift, we performed simulations of local ancestry under a model of neutral evolution using the SELAM software (Corbett-Detig and Jones 2016). We modeled a single-pulse of admixture in a Chilean population with global European, Native American, and African ancestry proportions of 0.521, 0.441, and 0.037, respectively, according to our LAMP-LD estimates. Admixture took place *T* = 10, 12, or 15 generations ago. These values of *T* were chosen because admixture among Chileans began in the 16^th^ century (about 14–15 generations ago) (Encina 1993), but the average time of admixture among Chileans was previously estimated to be 10 generations ago (Eyheramendy et al. 2015). We explored samples of size *N* = 93, 185, or 370 diploid individuals. For the case of 185 individuals (the size of the real sample), we simulated sexual populations (64 females and 121 males; matching the sexes of the real individuals), whereas for the other sample sizes, we simulated only hermaphroditic populations. We numerically estimated three effective population sizes (*N*_e_) based on the values of *T*, obtaining *N*_e_ = 4,500 at *T* = 10, *N*_e_ = 6,000 at *T* = 12, and *N*_e_ = 7,000 at *T* = 15 (see Materials and Methods). We simulated scenarios of neutral evolution under different combinations of *N*, *T*, and *N*_e_. For each scenario, we calculated *t*-test *P* values for the SNP with maximal European ancestry deviations (Table 1). No *P* value achieved the significant threshold of *P *<* *10^−5^ (Bhatia et al. 2014). Hence, our results indicate that the extreme deviations in European local ancestry observed in our Chilean population are not due to genetic drift and are consistent with an effect of PAS.

### Genomic Context and Functional Annotations of Selected Variants

**Fig. 4.**
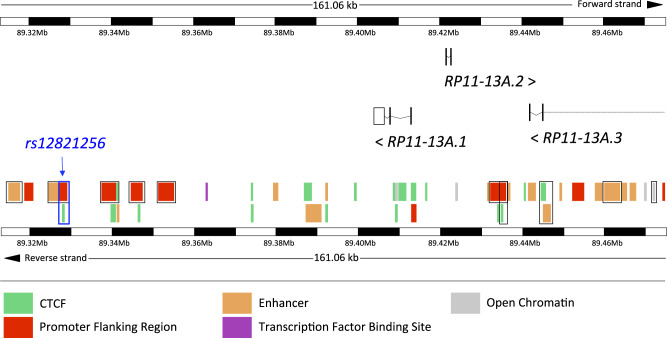
Genomic context of region in chromosome 12 affected by PAS. The image includes chromosome bands intercalated between black and white blocks with 10 kb of size, as well as several—sometimes overlapping—regulatory elements. The blue box encloses the regulatory region embracing a TFBS, a PFR, and a CTCF-BS that harbors *rs12821256.* Black boxes enclose enhancers, promoter-flanking regions, CTCF-BSs, and open chromatin regions associated by SNPs affected by PAS. Shown are the structures of *RP11-13A1.1*, *RP11-13A1.2*, and *RP11-13A1.3*, as well as the orientation of their coding sequences on the reference genome (“>” and “<” represent forward [5′–3′] and reverse [3′–5′] strands, respectively). Due to space limitations, we show the subregion with physical coordinates 12:89313336–89474394 instead of the whole region with coordinates 12:89193164–89556437. The image was adapted from an image retrieved from Ensembl.

We queried functional annotations of the 85 selected variants using the VEP web tool from Ensembl (McLaren et al. 2016). The strongest PAS hits (*P *=* *2.4×10^−7^) were 65 variants associated with the lncRNA *RP11-13A1.1*, the processed pseudogene *RP11-13A1.2*, the lncRNA *RP11-13A1.3* as well as several regulatory regions, including enhancers, promoter-flanking regions (PFR), CTCF-binding sites (CTCF-BS)—regions that binds CTCF, the insulator protein that demarcates open and closed chromatin (McLaren et al. 2016)—and an open chromatin region (supplementary table 1, Supplementary Material online). Interestingly, *RP11-13A1.1* has been implicated in host immune defense against infectious fungal pathogens (Riege et al. 2017) (see Discussion). One variant, *rs12821256*, maps a transcription factor binding site (TFBS) that overlaps a PFR as well as a CTCF-BS (supplementary table 1, Supplementary Material online). Figure 4 shows the detailed genomic context of *rs12821256* and *RP11-13A1.1*, highlighting associated regulatory elements undergoing PAS. Supplementary figure 1, Supplementary Material online, shows an expanded image of the whole chromosomal region.

To evaluate the deleteriousness potential of selected variants, we retrieved CADD scores from VEP. CADD scores integrate multiple annotations into a single metrics. Variants with CADD >15 are within the top 5% variants with the highest deleteriousness potential across the genome (Rentzsch et al. 2019). We found that some variants located in PFRs, CTCF-BSs, a TFBS, and one inter-PFR intergenic region have unusually high CADD scores (14.7–18.9), including the A, C, and G alleles of *rs12821256* (supplementary table 1, Supplementary Material online).

We queried the HaploReg tool (Ward and Kellis 2012) for eQTL associations of the regulatory variants with high CADD scores as well as for other epigenetic marks. Interestingly, *rs12821256* is an eQTL associated with the expression of *SUGT1* (*P *=* *2.6×10^−6^) and *SLC38A2* (*P *=* *2×10^−6^) in blood. In addition, *rs12821256* has promoter histone marks in skin cells (epidermal kerinocyte primary cells) and enhancer histone marks in 14 diverse tissues. We next queried the GWAS Catalog (Welter et al. 2014) to identify associations between selected genes and phenotypes. We found that significant GWAS associations have been reported between *rs12821256* and hair color (*rs12821256-T*; *P *=* *1×10^−100^) (Hysi et al. 2018) as well as with light versus dark hair color (*rs12821256-C*; *P *=* *2×10^−308^) (Morgan et al. 2018). We did not find known associations for *RP11-13A1.2* or *RP11-13A1.3*. Moreover, variants in *SUGT1* have been GWAS-associated with asthma (*P *=* *7×10^−12^) and respiratory system disease (*P *=* *2×10^−14^). Further, we found associations between *SLC38A2* variants and hair color (*P *=* *2×10^−11^) as well as lean body mass (*P *=* *1×10^−15^). Remarkably, *rs12821256* has been reported under strong selection in Europeans and East Asians (see Discussion).

## Discussion

The emergence of vast human population genetic data and the development of advanced analytical methods have enabled the detection of genetic variants involved in adaptation and complex diseases across diverse worldwide populations (Vitti et al. 2013; Lowe and Reddy 2015; Nielsen et al. 2017). Most of these studies have focused on African, European, and Asian populations. However, populations with Native American ancestry have been largely underrepresented (Cheng et al. 2019). This is due in part to: 1) a lack of publicly available genomes from several Native American populations and their admixed Latino descendants; 2) the technical difficulty in detecting ancestry-specific genetic factors in admixed populations with different continental ancestries and complex demographic histories, such as Latinos. In contrast to Peruvians, Colombians, Mexicans, and Puerto Ricans, for which there are publicly available whole-genome sequencing data (1000 Genomes Project Consortium et al. 2015), this is not the case for Chileans.

Chileans trace their genetic ancestry to Spaniards who admixed with Mapuche and Aymara Native Americans during the Columbian Exchange. On an average, Chileans have European, Native American, and African proportions of 0.52, 0.45, and 0.03, respectively (Eyheramendy et al. 2015). In addition, on an average, Chileans have 0.32 Mapuche and 0.11 Andean proportions (Andean proportion considered as the sum of the related Aymara, Quechua, Colla, and Southern Peru Andean proportions) (Chacon-Duque et al. 2018). However, ancestry and subancestry proportions vary considerably along Chile’s north–south geographic axis as well as among socio-economic groups. Although in the north Chileans have higher proportions of Aymara-related ancestry, in the south the main Native American subancestry is the Mapuche (Verdugo et al. 2020). In the present study, we estimated genetic relationships between admixed Chileans and several European and Native American populations. We analyzed a population from the Atacama Desert of Northern Chile (Vicuna et al. 2019). We found that this population has higher Aymara ancestry and lower Mapuche ancestry (25.9% vs. 20.7%, respectively; *K* = 5) than other more geographically heterogeneous Chilean samples with predominant Mapuche ancestry (Chacon-Duque et al. 2018). Therefore, our estimates from the admixed Atacama population are in agreement with a scenario of higher gene flow between Europeans and Andean populations in Northern Chile but lower admixture with the Mapuche from Central-Southern Chile. Of note, our PCs and global ancestry results show that the native Mapuche form a differentiated cluster when compared with the other Native American populations (*K* = 5–8). This is probably due to at least two effects resulting in a very low genetic diversity: 1) The Mapuche suffered a ∼95% decrease in their effective population size following the European colonization, based on an analysis that used imputed SNP array data from Mapuche genomes (Lindo et al. 2018); 2) The Mapuche individuals used in the present study were sampled in the small and relatively isolated Huapi Island and are expected to have extremely high-genetic relatedness. Indeed, 2 of the 11 original samples show second-degree relationships (IBD = 0.23–0.25) (Vidal et al. 2019); Unfortunately, these are the only available whole-genome sequences from modern Mapuche.

In the present study, we analyzed how gene flow from Europeans into Native Americans contributed adaptive genetic variation through PAS to admixed Chileans. We found that the strongest signals are associated with genes previously involved in pigmentation, thermogenesis, and immune defense against pathogens (Guenther et al. 2014; Yang et al. 2018), all of them phenotypes under strong selection in diverse human populations (Karlsson et al. 2014; Amorim et al. 2015; Yang et al. 2018). In relation to pigmentation, populations with dark skin tend to be protected against the lower folic acid levels induced by UV light, whereas light skin is an adaptation to maintain proper vitamin D levels (Deng and Xu 2018). Adaptation acting on thermogenesis enabled the Inuit as well as ancient Native American populations to adapt to extremely cold climates (Amorim et al. 2015, 2017; Fumagalli et al. 2015). Adaptation to dangerous infectious pathogens has occurred in response to the high mortality produced by them (Karlsson et al. 2014).

After the Columbian exchange, European colonization of the Americas resulted in the exposure of immigrants and natives to novel selective pressures. For example, many infectious diseases brought in by Europeans decimated native populations. These include smallpox, measles, influenza, and several others (Cook 1999; Waldman and Braun 2009). In contrast, there is little evidence supporting a role of endemic American diseases in the mortality of European settlers. Perhaps the strongest claim is syphilis, which caused a devastating pandemic in Europe that began in 1495, right after Columbus arrived to the United States. However, whether or not syphilis is endemic of the Americas is still subject of debate (de Melo et al. 2010). Arguably, to cope with the stronger selective pressures exerted by European infectious diseases, it is expected that the Latino admixed descendants of Native Americans and Europeans underwent PAS due to adaptive European genetic variation related with the immune response against pathogens brought by Europeans.

Our results show that one target undergoing PAS among Chileans is the *RP11-13A1.1* lncRNA. lncRNAs are an important class of noncoding RNAs that regulate gene expression through diverse mechanisms (Marchese et al. 2017). Interestingly, a transcriptome-wide study on lncRNAs following pathogen infection showed that *RP11-13A1.1* was strongly upregulated in monocytes upon infection of the fungal pathogens *Candida albicans* and *Aspergillus fumigatus*, but not upon infection of the bacteria *Escherichia coli*. Hence, the authors suggested that this lncRNA is a marker specific to fungal—but not bacterial—infections (Riege et al. 2017). Yet, a recent study showed that *RP11-13A1.1* was significantly downregulated in monocytes isolated from patients with Q fever, an infection caused by the zoonotic bacterium *Coxiella burnetii* (Raijmakers et al. 2019), suggesting that this lncRNA may affect the immune response against nonfungal infections as well. In addition, *RP11-13A1.1* was significantly and differentially upregulated in CD4^+^ T-cells of obese children with asthma compared with normal-weight children. The authors hypothesized that a possible role of T-cells during this condition is to increase neutrophilic airway inflammation among obese asthmatic children (Rastogi et al. 2018). Neutrophils are important players in the innate and adaptive immune systems, are the primary defense line against infection and one of the key cell types involved in initiation of the inflammatory response (Rosales et al. 2017).

Another relevant hit undergoing PAS is *rs12821256*, which maps an enhancer that regulates *KITLG* gene expression. *KITLG* encodes a ligand for the KIT receptor tyrosine kinase and regulates skin as well as hair pigmentation through the production of melanin pigments (Guenther et al. 2014; Yang et al. 2018). The molecular function of *rs12821256* in relation with hair pigmentation has been characterized in detail (Guenther et al. 2014). Trangenic mice carrying two *rs12821256-G* allele copies are significantly lighter than mice carrying two copies of the *rs12821256-A* ancestral allele, and also show significantly decreased *KITLG* expression in skin cells. Among humans, the blond-associated *rs12821256-A > G* substitution is prevalent in northern Europeans, but it is almost absent among Africans and Asians (Guenther et al. 2014). In addition, *rs12821256* has a significant effect on skin color in African–Americans (Miller et al. 2007). Further, *rs12821256* has been targeted by positive selection in Europeans (Grossman et al. 2010) and East Asians (Sturm and Duffy 2012; Yang et al. 2018). Moreover, other *KITLG* variants have been associated with skin color in the Han Chinese. Indeed, *KITLG* has undergone recurrent selection in European and East Asian populations (Williamson et al. 2007), possibly due to its adaptive effects on pigmentation and low temperatures at high latitude regions (Yang et al. 2018).

Besides its role in pigmentation and thermogenesis, *rs12821256* is also an eQTL for *SUGT1* expression in blood. *SUGT1* is a highly conserved gene among humans and mammals—including humans–, and is indispensable for the activity of inflammasomes (Mayor et al. 2007). These are a specialized group of intracellular sensors that are essential components of the host innate immune system against microbial and cellular insults, including those that occur in autoinflammatory diseases (Tartey and Kanneganti 2020). *SUGT1* variants have been significantly GWAS-associated with respiratory diseases with a strong inflammatory component, such as asthma. Noteworthy, higher proportions of Mapuche ancestry among admixed Chileans have been significantly associated with increase mortality rates due to asthma (Lorenzo Bermejo et al. 2017). Thus, *rs12821256* could also have contributed to PAS due to its regulatory effects over *SUGT1*. This might have been relevant in the context of the inflammatory response that occurred during asthma or other chronic inflammatory diseases in Chilean individuals under genetic risk during the Columbian Exchange.

Due to the pleiotropic associations between *rs12821256* and *KITLG* with pigmentation, thermogenesis, and expression levels of immune genes, it is difficult to identify the selective pressure that drove *rs12821256* into PAS. However, because the role of *KITLG* in adaptation related to skin pigmentation (Williamson et al. 2007; Grossman et al. 2010; Yang et al. 2018) and cold climate (Yang et al. 2018) has already been characterized in Eurasians, the most parsimonious hypothesis seems to be that *KITLG* variants may have been advantageous for Chileans due to similar reasons. Some observations support this hypothesis. 1) UV radiation ranges from extremely high in Northern Chile to extremely low in Southern Chile, and the highest levels of surface UV irradiance in the world have been measured in the Atacama Desert (Cordero et al. 2018). 2) There is high temperature variation along the 4,270 km north–south axis of Chile, and a big fraction of this geographical range—including the Patagonia region, where the Mapuche lived for centuries—shows very low temperatures (Miller 1976). It seems unlikely that *KITLG* variants were selected through sexual selection due to mating preferences for lighter hair color, since at least in Europeans, selection on hair color is mostly a pleiotropic effect of *KITLG* being selected for tolerance to climate and UV radiation (Stern et al. 2020).

Regarding *RP11-13A1.1*, we speculate that it may have contributed to immune defense against European infectious pathogens during the Columbian Exchange. This is because of two reasons. 1) The regulation of *RP11-13A1.1* has been well characterized in the context of immune defense against pathogens. 2) European infectious pathogens were an extreme selective pressure for Native American populations. However, it is also possible that *RP11-13A1.1* variants were hitchhiked into high frequency by *KITLG*-associated variants affected by PAS.

In conclusion, European variants of the haplotype in chromosome 12 underwent PAS most likely due to pleiotropic advantages that were relevant for Chileans following the Columbian Exchange. 

## Supplementary Material

evaa136_Supplementary_Data
