# Supplementary material for: Postadmixture Selection on Chileans Targets Haplotype Involved in Pigmentation, Thermogenesis and Immune Defense against Pathogens
Source: Genome Biol Evol. 2020 Jul 2;12(8):1459–70. doi: 10.1093/gbe/evaa136 (PMC7487163; doi:10.1093/gbe/evaa136)
Supplement: evaa136_Supplementary_Data [file evaa136_supplementary_data.pdf]

SUPPLEMENTARY FILES

Supplementary Figures

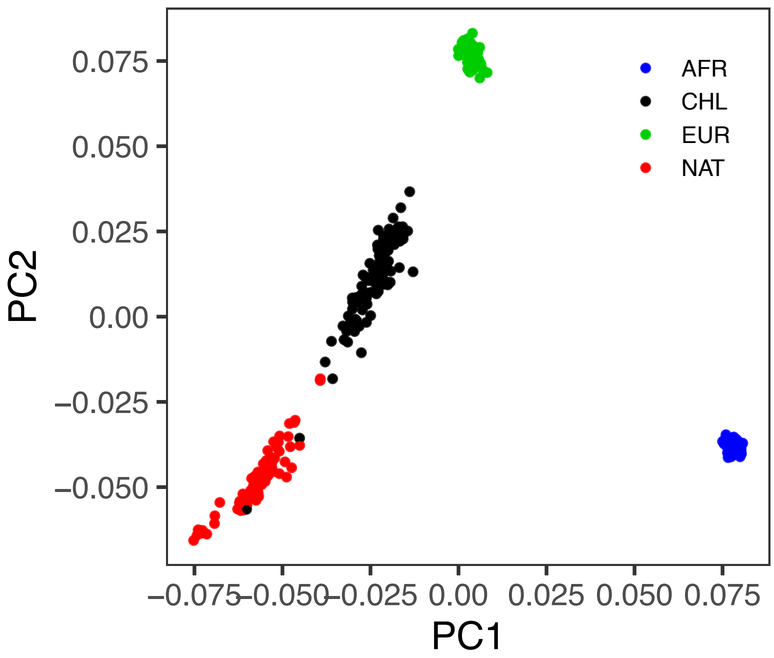

Supplementary Figure 1. Effect of sample size on PCA from Figure 1A. PC1 vs PC2 of 99 Chilean individuals together with 99 Europeans (IBS), 99 Native Americans (AYM, QUE, MAY, NAH, MAP) and 99 Africans (YRI).

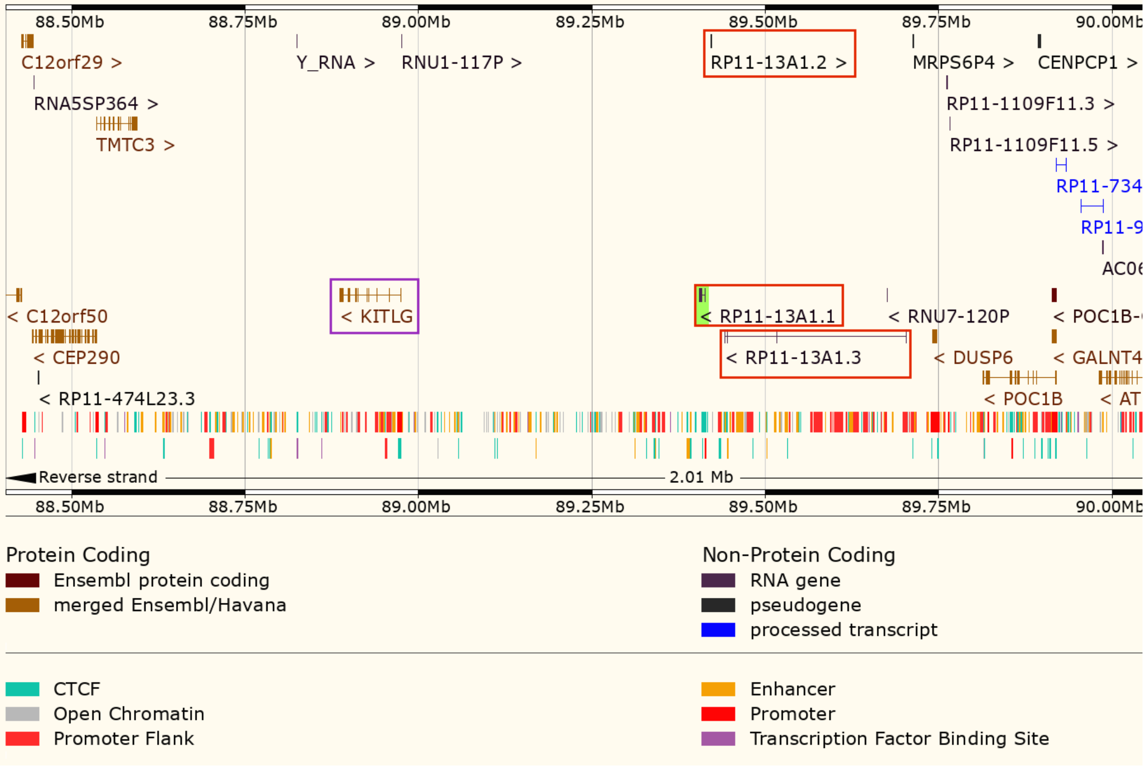

Supplementary Figure 2. Genetic context of region from chromosome 12 undergoing PAS. *RP11-13A1.1*, *RP11-13A1.2* and *RP11-13A1.3* are shown within red rectangles. The coordinates of the region under PAS are 12:89193164-89556437. However, we show a broader region to capture the nearby *KITLG* gene (shown within a purple rectangle) as well as other genetic elements. This image was retrieved from Ensembl GRCh37.

Supplementary Tables

SNP ID	POSITION	CHR	EUR ANC	P-VALUE	CONSEQ	GENE	BIOTYPE	CADD
rs17016050-A	89193164	12	0.6486	2.40E-07	IG	-	-	7.571
rs17016050-T	89193164	12	0.6486	2.40E-07	IG	-	-	6.953
rs11105060-C	89195117	12	0.6486	2.40E-07	IG	-	-	5.129
rs1381908-T	89197233	12	0.6486	2.40E-07	IG	-	-	1.282
rs11105062-G	89198983	12	0.6486	2.40E-07	IG	-	-	1.852
rs454833-G	89200595	12	0.6486	2.40E-07	IG	-	-	0.752
rs17016079_A	89204722	12	0.6486	2.40E-07	IG	-	-	1.764
rs1462978-G	89204842	12	0.6486	2.40E-07	IG	-	-	1.189
rs17016082-A	89205024	12	0.6486	2.40E-07	IG	-	-	0.313
rs1398303-A	89205094	12	0.6486	2.40E-07	IG	-	-	2.139
rs11105065-C	89207726	12	0.6486	2.40E-07	IG	-	-	0.271
rs11105066-T	89207924	12	0.6486	2.40E-07	IG	-	-	1.503
rs506900-A	89224640	12	0.6486	2.40E-07	IG	-	-	0.309
rs1512419-A	89227133	12	0.6486	2.40E-07	IG	-	-	11.21
rs11105077-C	89235211	12	0.6486	2.40E-07	IG	-	-	6.925
rs17016116-A	89255080	12	0.6486	2.40E-07	IG	-	-	1.06
rs10506959-T	89255723	12	0.6486	2.40E-07	IG	-	-	4.764
rs10506960-C	89255847	12	0.6486	2.40E-07	IG	-	-	0.208
rs10506960-T	89255847	12	0.6486	2.40E-07	IG	-	-	0.221
rs11609776-G	89258855	12	0.6486	2.40E-07	IG	-	-	3.219
rs11609776-T	89258855	12	0.6486	2.40E-07	IG	-	-	2.956
rs7298581-A	89258874	12	0.6486	2.40E-07	IG	-	-	1.708
rs17350615-C	89259060	12	0.6486	2.40E-07	IG	-	-	3.693
rs10858795-C	89261571	12	0.6486	2.40E-07	REG	-	CTCF_BS	1.69
rs10858795-C	89261571	12	0.6486	2.40E-07	IG	-	-	1.69
rs2045808-T	89275759	12	0.6486	2.40E-07	IG	-	-	1.145
rs1512424-C	89292833	12	0.6486	2.40E-07	IG	-	-	1.007
rs1512424-G	89292833	12	0.6486	2.40E-07	IG	-	-	0.858
rs12303900-G	89299016	12	0.6486	2.40E-07	IG	-	-	8.92
rs386799-A	89301928	12	0.6486	2.40E-07	IG	-	-	1.936
rs386799-C	89301928	12	0.6486	2.40E-07	IG	-	-	1.338
rs386799-T	89301928	12	0.6486	2.40E-07	IG	-	-	1.419
rs198639-A	89313894	12	0.6486	2.40E-07	IG	-	-	1.4
rs198638-C	89314180	12	0.6486	2.40E-07	IG	-	-	4.414
rs11105106-A	89315042	12	0.6486	2.40E-07	REG	-	enhancer	8.189
rs11105106-A	89315042	12	0.6486	2.40E-07	IG	-	-	8.189
rs17815185-A	89325208	12	0.6486	2.40E-07	REG	-	enhancer	0.015
rs17815185-T	89325208	12	0.6486	2.40E-07	REG	-	enhancer	0.01
rs17815185-A	89325208	12	0.6486	2.40E-07	IG	-	-	0.015
rs17815185-T	89325208	12	0.6486	2.40E-07	IG	-	-	0.01
rs12821256-A	89328335	12	0.6486	2.40E-07	REG	-	PFR	14.72
rs12821256-C	89328335	12	0.6486	2.40E-07	REG	-	PFR	14.8
rs12821256-G	89328335	12	0.6486	2.40E-07	REG	-	PFR	14.69
rs12821256-A	89328335	12	0.6486	2.40E-07	REG	-	CTCF_BS	14.72
rs12821256-C	89328335	12	0.6486	2.40E-07	REG	-	CTCF_BS	14.8

SNP ID	POSITION	CHR	EUR ANC	P-VALUE	CONSEQ	GENE	BIOTYPE	CADD
rs12821256-G	89328335	12	0.6486	2.40E-07	REG	-	CTCF_BS	14.69
rs12821256-A	89328335	12	0.6486	2.40E-07	TFBS	-	-	14.72
rs12821256-C	89328335	12	0.6486	2.40E-07	TFBS	-	-	14.8
rs12821256-G	89328335	12	0.6486	2.40E-07	TFBS	-	-	14.69
rs12821256-A	89328335	12	0.6486	2.40E-07	IG	-	-	14.72
rs12821256-C	89328335	12	0.6486	2.40E-07	IG	-	-	14.8
rs12821256-G	89328335	12	0.6486	2.40E-07	IG	-	-	14.69
rs661114-A	89337963	12	0.6486	2.40E-07	REG	-	PFR	0.186
rs661114-A	89337963	12	0.6486	2.40E-07	IG	-	-	0.186
rs10858804-G	89339129	12	0.6486	2.40E-07	REG	-	PFR	6.132
rs10858804-G	89339129	12	0.6486	2.40E-07	IG	-	-	6.132
rs12298351-T	89340112	12	0.6486	2.40E-07	REG	-	PFR	17.75
rs12298351-T	89340112	12	0.6486	2.40E-07	REG	-	CTCF_BS	17.75
rs12298351-T	89340112	12	0.6486	2.40E-07	IG	-	-	17.75
rs114873-A	89340293	12	0.6486	2.40E-07	REG	-	PFR	17.96
rs114873-A	89340293	12	0.6486	2.40E-07	REG	-	CTCF_BS	17.96
rs114873-A	89340293	12	0.6486	2.40E-07	IG	-	-	17.96
rs390594-T	89345231	12	0.6486	2.40E-07	REG	-	PFR	1.01
rs390594-T	89345231	12	0.6486	2.40E-07	IG	-	-	1.01
rs10161354-G	89345489	12	0.6486	2.40E-07	REG	-	PFR	2.141
rs10161354-G	89345489	12	0.6486	2.40E-07	IG	-	-	2.141
rs11105118-C	89349282	12	0.6486	2.40E-07	IG	-	-	18.94
rs714561-A	89354967	12	0.6486	2.40E-07	REG	-	PFR	8.265
rs714561-A	89354967	12	0.6486	2.40E-07	IG	-	-	8.265
rs10858809-G	89365305	12	0.6486	2.40E-07	IG	-	-	8.199
rs2058343-G	89366763	12	0.6486	2.40E-07	IG	-	-	1.55
rs7969593-A	89373308	12	0.6486	2.40E-07	IG	-	-	4.689
rs10858811-A	89381894	12	0.6486	2.40E-07	IG	-	-	0.573
rs4842479-T	89385017	12	0.6486	2.40E-07	IG	-	-	2.155
rs7976001-C	89400810	12	0.6486	2.40E-07	DS	RP11-13A1.1	lincRNA	8.616
rs1012421-G	89425822	12	0.6486	2.40E-07	DS	RP11-13A1.2	PP	10.36
rs10858818-C	89426732	12	0.6486	2.40E-07	DS	RP11-13A1.2	PP	0.825
rs12311684-G	89435177	12	0.6486	2.40E-07	DS	RP11-13A1.3	lincRNA	3.563
rs12311684-G	89435177	12	0.6486	2.40E-07	REG	-	PFR	3.563
rs12311684-G	89435177	12	0.6486	2.40E-07	REG	-	CTCF_BS	3.563
rs11611632-T	89445539	12	0.6486	2.40E-07	IN-NCT	RP11-13A1.3	lincRNA	1.582
rs11611632-T	89445539	12	0.6486	2.40E-07	REG	-	CTCF_BS	1.582
rs11611632-T	89445539	12	0.6486	2.40E-07	REG	-	enhancer	1.582
rs10506961-A	89462175	12	0.6486	2.40E-07	IN-NCT	RP11-13A1.3	lincRNA	0.782
rs10506961-A	89462175	12	0.6486	2.40E-07	REG	-	enhancer	0.782
rs10506962-C	89462738	12	0.6486	2.40E-07	IN-NCT	RP11-13A1.3	lincRNA	5.101
rs10506962-C	89462738	12	0.6486	2.40E-07	REG	-	enhancer	5.101
rs10506963-A	89462969	12	0.6486	2.40E-07	IN-NCT	RP11-13A1.3	lincRNA	0.708
rs10506963-T	89462969	12	0.6486	2.40E-07	IN-NCT	RP11-13A1.3	lincRNA	0.514
rs10506963-A	89462969	12	0.6486	2.40E-07	REG	-	enhancer	0.708

SNP ID	POSITION	CHR	EUR ANC	P-VALUE	CONSEQ	GENE	BIOTYPE	CADD
rs10506963-T	89462969	12	0.6486	2.40E-07	REG	-	enhancer	0.514
rs10777150-C	89463314	12	0.6486	2.40E-07	IN-NCT	RP11-13A1.3	lincRNA	4.373
rs10777150-C	89463314	12	0.6486	2.40E-07	REG	-	enhancer	4.373
rs12820818-T	89470891	12	0.6486	2.40E-07	IN-NCT	RP11-13A1.3	lincRNA	8.421
rs10858822-A	89471403	12	0.6486	2.40E-07	IN-NCT	RP11-13A1.3	lincRNA	4.423
rs6538168-A	89471844	12	0.6486	2.40E-07	IN-NCT	RP11-13A1.3	lincRNA	3.398
rs11105167-C	89471928	12	0.6486	2.40E-07	IN-NCT	RP11-13A1.3	lincRNA	6.12
rs11105167-C	89471928	12	0.6486	2.40E-07	REG	-	OCR	6.12
rs11105168-C	89473626	12	0.6486	2.40E-07	IN-NCT	RP11-13A1.3	lincRNA	3.382
rs11105168-T	89473626	12	0.6486	2.40E-07	IN-NCT	RP11-13A1.3	lincRNA	3.48
rs11105170-G	89475577	12	0.6486	2.40E-07	IN-NCT	RP11-13A1.3	lincRNA	6.39
rs11105170-G	89475577	12	0.6486	2.40E-07	REG	-	PFR	6.39
rs11105171-G	89475779	12	0.6486	2.40E-07	IN-NCT	RP11-13A1.3	lincRNA	1.213
rs11105171-G	89475779	12	0.6486	2.40E-07	REG	-	PFR	1.213
rs12426442-C	89478882	12	0.6486	2.40E-07	IN-NCT	RP11-13A1.3	lincRNA	9.616
rs12426442-C	89478882	12	0.6486	2.40E-07	REG	-	PFR	9.616
rs11105173-C	89487797	12	0.6486	2.40E-07	IN-NCT	RP11-13A1.3	lincRNA	3.094
rs11834560-T	89488629	12	0.6486	2.40E-07	IN-NCT	RP11-13A1.3	lincRNA	0.734
rs1949536-A	89490667	12	0.6486	2.40E-07	IN-NCT	RP11-13A1.3	lincRNA	1.292
rs1879205-C	89490962	12	0.6486	2.40E-07	IN-NCT	RP11-13A1.3	lincRNA	3.384
rs11105174-C	89492878	12	0.6486	2.40E-07	IN-NCT	RP11-13A1.3	lincRNA	4.814
rs7962036-T	89496725	12	0.6432	7.50E-07	IN-NCT	RP11-13A1.3	lincRNA	2.199
rs939283-T	89497841	12	0.6432	7.50E-07	IN-NCT	RP11-13A1.3	lincRNA	5.835
rs939284-G	89498294	12	0.6432	7.50E-07	IN-NCT	RP11-13A1.3	lincRNA	0.916
rs939284-T	89498294	12	0.6432	7.50E-07	IN-NCT	RP11-13A1.3	lincRNA	0.822
rs3926062-A	89504012	12	0.6432	7.50E-07	IN-NCT	RP11-13A1.3	lincRNA	9.048
rs11833629-T	89506544	12	0.6432	7.50E-07	IN-NCT	RP11-13A1.3	lincRNA	1.607
rs1356774-C	89510867	12	0.6432	7.50E-07	IN-NCT	RP11-13A1.3	lincRNA	6.407
rs1356774-G	89510867	12	0.6432	7.50E-07	IN-NCT	RP11-13A1.3	lincRNA	5.986
rs12369825-T	89512646	12	0.6432	7.50E-07	IN-NCT	RP11-13A1.3	lincRNA	1.554
rs1519959-G	89515138	12	0.6432	7.50E-07	IN-NCT	RP11-13A1.3	lincRNA	5.684
rs1519959-G	89515138	12	0.6432	7.50E-07	REG	-	enhancer	5.684
rs17584017-T	89515681	12	0.6432	7.50E-07	IN-NCT	RP11-13A1.3	lincRNA	2.308
rs35388619-G	89520000	12	0.6432	7.50E-07	IN-NCT	RP11-13A1.3	lincRNA	2.817
rs35388619-G	89520000	12	0.6432	7.50E-07	REG	-	enhancer	2.817
rs1519963-A	89520977	12	0.6432	7.50E-07	IN-NCT	RP11-13A1.3	lincRNA	5.163
rs1519963-A	89520977	12	0.6432	7.50E-07	REG	-	enhancer	5.163
rs1983374-A	89525052	12	0.6432	7.50E-07	IN-NCT	RP11-13A1.3	lincRNA	4.082
rs41354950-C	89527591	12	0.6432	7.50E-07	IN-NCT	RP11-13A1.3	lincRNA	6.068
rs7138825-A	89528992	12	0.6432	7.50E-07	IN-NCT	RP11-13A1.3	lincRNA	6.813
rs7138825-A	89528992	12	0.6432	7.50E-07	REG	-	enhancer	6.813
rs1996640-A	89535861	12	0.6432	7.50E-07	IN-NCT	RP11-13A1.3	lincRNA	3.787
rs17016437-C	89535970	12	0.6432	7.50E-07	IN-NCT	RP11-13A1.3	lincRNA	7.204
rs17352485-A	89551603	12	0.6324	6.25E-06	IN-NCT	RP11-13A1.3	lincRNA	0.228
rs17352485-T	89551603	12	0.6324	6.25E-06	IN-NCT	RP11-13A1.3	lincRNA	0.316

SNP ID	POSITION	CHR	EUR ANC	P-VALUE	CONSEQ	GENE	BIOTYPE	CADD
rs7957097-A	89553345	12	0.6324	6.25E-06	IN-NCT	RP11-13A1.3	lincRNA	0.26
rs7957097-T	89553345	12	0.6324	6.25E-06	IN-NCT	RP11-13A1.3	lincRNA	0.185
rs1356773-A	89555278	12	0.6324	6.25E-06	IN-NCT	RP11-13A1.3	lincRNA	3.056
rs1356773-C	89555278	12	0.6324	6.25E-06	IN-NCT	RP11-13A1.3	lincRNA	3.335
rs11105194-T	89556437	12	0.6324	6.25E-06	IN-NCT	RP11-13A1.3	lincRNA	3.631

Supplementary Table 1. Variants with enrichment in mean local European ancestry. Variants with significant deviations in the mean local European ancestry over the genome-wide mean among admixed Chileans. Shown are the corresponding SNP ID, physical position, chomosome, mean European local ancestry, P-value of the t-test, SO consequence type, associated gene, biotype and CADD-PHRED score. Abbreviations: CTCF-BS: CTCF binding site; IG: intergenic; DS: downstream; IN: intron; IN-NCT: intron/non-coding transcript; lincRNA: long noncoding RNA; OCR: open chromatin region; PP: processed pseudogene; REG: regulatory region; TFBS: transcription factor binding site. Shown are consequence types per variant allele.

N	Ne	T	Mean	Var	Min	Max	P-Value
93	4,500	10	0.5033	0.0028	0.2541	0.654	0.00674
185	4,500	10	0.4951	0.0027	0.2796	0.624	0.02506
185*	4,500	10	0.4938	0.0023	0.3162	0.665	0.00159
370	4,500	10	0.4962	0.0022	0.3581	0.628	0.01185
93	6,000	12	0.5272	0.0015	0.4162	0.638	0.00184
185	6,000	12	0.5294	0.0016	0.4140	0.667	0.00018
185*	6,000	12	0.5161	0.0009	0.4351	0.649	0.00002
370	6,000	12	0.5179	0.0005	0.4473	0.596	0.00068
93	7,000	15	0.5211	0.0015	0.4054	0.686	0.00002
185	7,000	15	0.5246	0.0018	0.3978	0.677	0.00017
185*	7,000	15	0.5216	0.0010	0.4135	0.630	0.00041
370	7,000	15	0.5254	0.0007	0.4311	0.615	0.00015

Supplementary Table 2. Local European ancestry deviations under simulations of neutral evolution. Effect of sample size N (number of haploid chromosomes), effective population size Ne and time T since admixture (in number of generations) on local EUR ancestry deviations over the genome-wide EUR ancestry mean. Besides sexual populations labeled with an asterisk, all other simulated populations are hermaphroditic. Shown are the mean, variance, minimum and maximum of EUR ancestry for the locus exhibiting the highest deviation under each combination of N, Ne and T, as well as the corresponding t-test P-value.
